# Association between living arrangements, social support, and depression among middle-aged and older adults: a mediation analysis from the CHARLS survey

**DOI:** 10.3389/fpsyg.2025.1492495

**Published:** 2025-02-10

**Authors:** Fenghua Jin, Yixuan Hu

**Affiliations:** School of Foreign Languages, Renmin University of China, Beijing, China

**Keywords:** living arrangement, social support, depression, mediation analysis, CHARLS

## Abstract

**Introduction:**

Living alone was reported to be associated with a higher risk of depression. Social support may play a crucial role in mediating this association. However, data are limited.

**Methods:**

Data for wave 5 (2020) of the China Health and Retirement Longitudinal Study (CHARLS) were extracted. Associations between living arrangements and social support or depression were assessed by multivariable logistic regression models. Causal mediation analysis under a counterfactual framework was employed to evaluate the mediation effect of social support in the association between living arrangements and depression, which was performed by fitting two logistic regression models. The mediation effect is measured by the percentage mediated.

**Results:**

A total of 17,418 participants were included in this study, of which 208 (1.2%) lived alone. Compared to participants not living alone, those living alone were associated with a higher risk of low social support (10.6% vs. 3.9%; adjusted OR [aOR], 1.75; 95% CI, 1.10–2.80) and depression (15.4% vs. 7.2%; adjusted OR, 1.53; 95% CI, 1.02–2.28). Mediation analyses revealed that 2.7% (95% CI, −1.1% to 6.5%) of the relationship between living arrangements and depression was mediated by social support. Sensitivity analyses by varying definitions of living alone or limiting analysis in the elderly population yielded consistent results.

**Conclusions:**

Low social support did not mediate the association between living status and depression. Tailored strategies for improving living arrangements may needed to improve the mental health of living alone older adults.

## Introduction

China is experiencing rapid change, with a growing number of middle-aged and older adults facing the challenges of living alone due to urbanization, especially elderly adults (aged over 60), which account for over 18% of the total population in 2020 (Tu et al., 2022). This demographic shift has significant implications for the mental health of the population, particularly in terms of depression prevalence. Depression among older adults in China has emerged as a critical public health concern, with studies indicating high rates of depressive symptoms in this population (Zhang et al., 2019; Zhong et al., 2019, 2020; Ma et al., 2024).

The process of urbanization in China has led to a transformation of traditional family structures, resulting in an increasing number of middle-aged and older adults living independently from their children (Yang and Chen, 2019). This change in living arrangements has been associated with a higher risk of social isolation and loneliness (Zhong et al., 2018; Yuan et al., 2024), which are known risk factors for depression in later life (Park et al., 2017).

Research suggests that social support may play a crucial role in mediating the relationship between living alone and depression among middle-aged and older adults in China. Social support, which encompasses emotional, instrumental, and informational assistance from family members, friends, and community networks, has been shown to have protective effects against depression in various populations (Wang et al., 2018).

Given the complex interplay between living arrangements, social support, and mental health outcomes in China's aging population, it is essential to examine the potential mediating role of social support in the association between living alone and depression among middle-aged and older adults (Lu et al., 2019; Pei et al., 2022). This investigation aims to contribute to the growing body of literature on aging in China and inform public health interventions targeting the mental well-being of the middle-aged and older population.

## Methods

### Data source

The data for the current study were extracted from wave 5 of the China Health and Retirement Longitudinal Study (CHARLS). The detailed design of the CHARLS study has been reported elsewhere (Zhao et al., 2014). Briefly, The CHARLS study is harmonized with the Health and Retirement Study (HRS) in the USA (Zhong et al., 2019). The CHARLS is a national survey of a representative sample of Chinese residents aged 45 years and older, aiming to assess community residents' social, economic, and health circumstances. For the current study, we used the wave 5 data collected during 2020. Participants without spouses and children were excluded from this analysis.

### Study variables

#### Exposure, mediator, and outcome

The exposure is the living arrangements of the participants, which was classified as living alone or not living alone. Living alone was defined as equal to 182 days (100% of the first half year) to the question, “How many days did you live alone in the first half of this year?”. In addition, we also defined living alone as living alone equal to or larger than 146 days (80% of the first half year) in the sensitivity analysis. The mediator was social support, which was defined by the frequency of seeing or contacting children and activities in the last year, as children encompass a crucial role in the family unit. Participants with a frequency of seeing children equal to or less than once a month, contacting children equal to or less every 2 weeks, and having no activities in the last month were classified as low social support. The outcome was depression. Common symptoms of depression include feeling sad, lethargic, and fearful. Depression in CHARES was measured by the Chinese version of the 10-item Centre for Epidemiological Studies Depression Scale (CESD-10) (Cheng and Chan, 2005), a validated and widely used instrument for diagnosing depression in epidemiological studies. Each item was scored from 0 to 3, with the sum of 10 items scored between 0 and 30. Depression was defined as a CESD-10 score ≥20.

#### Potential covariables

The following variables collected in the CHARLS study were also assessed as potential covariates in the current study: age, sex, marital status (married; separated, divorced, or widowed), live location (city center or town center, combination zone between urban and rural areas, village and other), and self-reported chronic disease, including hypertension, dyslipidemia, diabetes, heart diseases, stomach or digestive disorders, and arthritis or rheumatism.

### Statistical analysis

#### Descriptive analysis

Data were summarized by means and standard deviations, or medians (interquartile ranges) for continuous variables, and frequencies along with percentages for categorical variables. Differences between living status groups were assessed by absolutely standard difference (ASD). An ASD ≥10% was considered to be significantly different in statistics and clinical (Austin, 2009).

#### Association analysis

The associations between living status and social support or depression were assessed by multivariable logistic regression models. Odds ratios and 95% CIs were reported. The covariates in the models were determined by a comprehensive consideration of the literature review, clinical expertise, and distribution between living status, which included age, sex, marital status, and self-reported chronic diseases.

#### Mediation analysis

**Figure 1:**
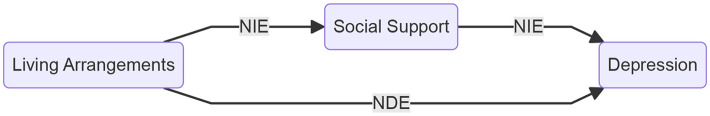
Directed acyclic graph for the mediation effect of social support. NDE, natural direct effect; NIE, natural indirect effect. Total effect = NDE + NIE.

We used a directed acyclic graph to illustrate the association of living status with social support and depression (Figure 1). We performed causal mediation analysis under a counterfactual framework, in which a clear definition of the mediation effect was provided under a general framework (Robins and Greenland, 1992; Pearl, 2001; Valeri and Vanderweele, 2013). Under this framework, the total effect (TE) was divided into two parts measured as OR: the natural direct effect (NDE) and the natural indirect effect (NIE). The NDE represented the direct effect of living arrangements on depression, while the NIE represented the effect of living arrangements on depression via low social support. The mediation effect is measured by percentage mediated (PM), computed as NIE/TE^*^100% on a log-transformed OR scale, which is the percentage of the TE that the mediator mediates (Vanderweele and Vansteelandt, 2010). We fitted two logistic regression models to calculate the mediation effect. One is the mediator model, a multivariable logistic regression model for low social support (mediator) conditional on living arrangements (exposure) and all study confounders. Another model is the outcome model, a multivariable logistic regression model for depression (outcome) conditional on living arrangements, social support, and all study confounders. Based on a comprehensive review of the literature, relevant medical knowledge from medical guidelines (Feng et al., 2019; Guideline Development Panel for the Treatment of Depressive Disorders, 2022; Qaseem et al., 2023), the data that CHARLS study collected, and the imbalances observed in Table 1, confounders examined in the causal mediation analysis were determined, which included demographics (age, sex) and disease history (overall disease, dyslipidemia, arthritis, or rheumatism).

**Table 1:** Demographics and covariates by living status.

**Variables**	**Living- alone (*N* = 208 [1.2%])**	**Not living- alone (*N* = 17210 [98.8%])**	ASD (%)^*^
Age	68.1 (10.5)	60.9 (9.5)	71.0
Age groups			50.8
<60 years	48 (23.1)	8,007 (46.5)	
≥60 years	160 (76.9)	9,203 (53.5)	
Sex			10.0
Male	86 (41.3)	7,971 (46.3)	
Female	122 (58.7)	9,239 (53.7)	
Marital Status			219.0
Married	26 (12.5)	14 856 (86.3)	
Separated, Divorced or Widowed	182 (87.5)	2,354 (13.7)	
Live location			7.2
Missing	0 (0.0)	3 (0.0)	
City Center or Town Center	46 (22.1)	4,060 (23.6)	
Combination Zone Between Urban and Rural Areas	22 (10.6)	2,101 (12.2)	
Village	139 (66.8)	11 028 (64.1)	
Other	1 (0.5)	18 (0.1)	
Chronic diseases	67 (32.2)	6,344 (36.9)	9.8
Hypertension	18 (8.7)	1,218 (7.1)	5.9
Dyslipidemia	11 (5.3)	1,422 (8.3)	11.9
Diabetes	12 (5.8)	766 (4.5)	6.0
Heart diseases	15 (7.2)	883 (5.1)	8.7
Stomach or digestive disorders	10 (4.8)	949 (5.5)	3.2
Arthritis or rheumatism	8 (3.8)	1,149 (6.7)	12.7

* ASD means absolutely standard difference; an ASD ≥10% indicates a significant difference in statistical and clinical.

All statistical analyses were performed using SAS statistical software version 9.4 (SAS Institute Inc.). We used a SAS macro named %ggBaseline to generate the descriptive tables more efficiently (Gu et al., 2018).

## Results

### Study population

**Figure 2:**
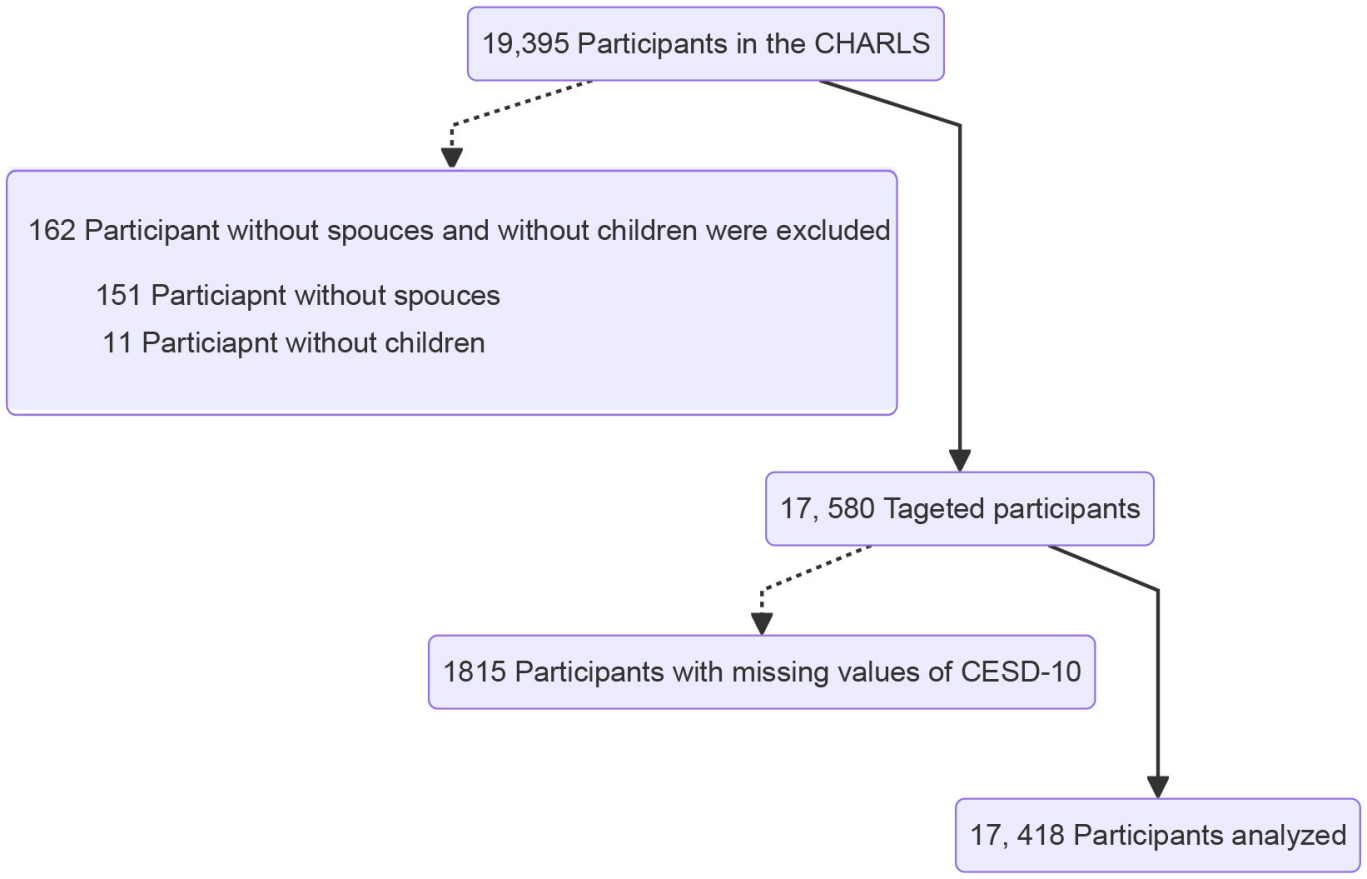
Patient identification chart. CHARLS, the China Health and Retirement Longitudinal Study; CESD-10, the Chinese version of the 10-item Centre for Epidemiological Studies Depression Scale.

The wave 5 (2020) of the CHARLS survey enrolled 19,395 participants. After excluding 162 participants without spouses and children and 1,815 participants with missing values of CESD-10, a total of 17,418 participants were analyzed in the current study. Of them, 208 (1.2%) participants lived alone (Figure 2).

### Characteristics of study participants

Living alone participants were older in age (68.1 vs. 60.9, ASD=71.0%), more female (58.7% vs. 53.7%, ASD=10.0%), and more separated, divorced, or widowed (87.5% vs. 13.7%, ASD=219.0%). However, living-alone participants had fewer self-reported chronic diseases, especially for dyslipidemia (5.3% vs. 8.3%, ASD=11.9%) and arthritis or rheumatism (3.8% vs. 6.7%, ASD=12.7%) (Table 1).

### Association between living arrangements, social support, and depression

Participants with low social support were observed in 10.6% (*n* = 22) and 3.9% (*n* = 668) of the living-alone participants and not living-alone participants, respectively. Depressions, as defined by a CESD-10 score ≥20, were seen in 15.4% (*n* = 32) and 7.2% (*n* = 1,234) of the participants among the two groups, respectively. After adjusting for covariates, living-alone participants had significantly higher odds of low social support (adjusted OR [aOR], 1.75; 95% CI, 1.10–2.80) and depression (aOR, 1.53; 95% CI, 1.02–2.28). In addition, low social support was associated with higher odds of depression (aOR,1.54; 95% CI, 1.20–1.98) (Table 2).

**Table 2:** Association between living arrangements, social support, and depression.

**Outcome**	**Factor**	**No. of patients**	**Outcome no. (%)**	**Unadjusted analysis**		**Adjusted analysis** ^*^	
				**Odds Ratio (95% CI)**	***P*** **Value**	**Odds Ratio (95% CI)**	***P*** **Value**
Low social support	Live alone						
	Yes	208	22 (10.6)	2.93 (1.87–4.59)	<0.001	1.75 (1.10–2.80)	0.02
	No	17,210	668 (3.9)	1.0 (Reference)		1.0 (Reference)	
Depression	Live alone						
	Yes	208	32 (15.4)	2.35 (1.61–3.45)	<0.001	1.53 (1.02–2.28)	0.04
	No	17,210	1,234 (7.2)	1.0 (Reference)		1.0 (Reference)	
Depression	Low social support						
	Yes	690	76 (11.0)	1.62 (1.26–2.07)	<0.001	1.54 (1.20–1.98)	<0.001
	No	16,728	1,190 (7.1)	1.0 (Reference)		1.0 (Reference)	

* Adjusted for age, sex, overall disease, dyslipidemia, arthritis or rheumatism.

### Mediation analysis

The odds ratio total effects, direct associations, and indirect associations of living status with depression were presented in Table 3. The indirect association via low social support implied that a 1% increase in the risk of depression (aOR 1.01; 95% CI, 1.00–1.02) would be observed on average. The proportion of the association between living arrangements and depression mediated by low social support was 2.7% (95% CI, −1.1% to 6.5%). We also calculated the estimates of direct and indirect associations by defining living alone as equal to or larger than 146 days. Results showed that social support mediated 1.8% (95% CI −0.1% to 3.7%) of the association between living arrangements and depression (Table 3). Sensitivity analysis by limiting the mediation analysis among ages over 60 years old population showed similar results mediated 1.5% (95% CI −3.21% to −6.3%; Table 3).

**Table 3:** Association of living status with depression mediated by social support.

**Variables**	**Unadjusted analysis**		**Adjusted analysis** ^*^	
	**Estimate (95%CI)**	***P***	**Estimate (95%CI)**	***P***
**Primary analysis**				
Odds ratio total effect	2.33 (1.44–3.23)	0.0034	1.51 (0.90–2.12)	0.0984
Odds ratio natural direct effect (NDE)	2.28 (1.41–3.15)	0.0041	1.50 (0.90–2.10)	0.1046
Odds ratio natural indirect effect (NIE)	1.03 (1.01–1.05)	0.0119	1.01 (1.00–1.02)	0.0950
Percentage mediated	4.4 (0.9 to 7.9)	0.0151	2.7 (–1.1 to 6.5)	0.1593
**Sensitivity analysis with a wider living-alone definition**				
Odds ratio total effect	2.00 (1.66–2.34)	<0.0001	1.46 (1.16–1.76)	0.0026
Odds ratio natural direct effect (NDE)	1.98 (1.64–2.31)	<0.0001	1.45 (1.15–1.75)	0.0029
Odds ratio natural indirect effect (NIE)	1.01 (1.01–1.02)	<0.0001	1.01 (1.00–1.01)	0.0029
Percentage mediated	2.7 (1.0 to 4.4)	0.0018	1.8 (–0.1 to 3.7)	0.0524
**Sensitivity analysis among ages over 60**				
Odds ratio total effect	1.76 (0.96–2.56)	0.0634	1.25 (0.66–1.85)	0.4030
Odds ratio natural direct effect (NDE)	1.74 (0.95–2.54)	0.0669	1.25 (0.66–1.85)	0.4089
Odds ratio natural indirect effect (NIE)	1.01 (0.990–1.03)	0.2459	1.00 (0.99–1.01)	0.4196
Percentage mediated	2.2 (–1.7 to 6.1)	0.2697	1.5 (–3.2 to −6.3)	0.0984

* Adjusted for age, sex, overall disease, dyslipidemia, arthritis or rheumatism.

## Discussion

In this national survey of a representative sample of Chinese residents aged 45 years and older, we found that living alone was associated with a higher risk of low social support, and also associated with a higher risk of depression. Low social support was associated with a higher risk of depression as well. However, results from mediation analysis revealed that the association between living arrangements and depression was not mediated by social support.

Previous studies in other countries, including Japan, Korea, the US, and China, also reported the association between living alone and depression (Fukunaga et al., 2012; Park et al., 2017; Pei et al., 2020; Hu et al., 2023; Miyake et al., 2023). Social support or social activities were reported to be a mediator of the association between living status and depression (Lu et al., 2019; Xie et al., 2023). However, our study revealed that social support did not mediate this association. Based on this study, interventions to lower the depression of older people should be more specific to improving their living arrangements instead of social support.

There are several potential reasons why social support did not mediate the association between living status and depression, which may include the heterogeneities with other studies in design and analysis. Of these potential reasons, the most fundamental reason may be that the association between living alone and depression was sufficiently strong, and the effect of social support was weak in both magnitude and significance (Gariepy et al., 2016).

Several strategies for improving living arrangements have been reported to be associated with improved depression status. For example, compared to living alone, living with others (Zissimopoulos and Thunell, 2020), having a pet (Miyake et al., 2023), or living alone but not eating alone (Tani et al., 2015), may mitigate feelings of loneliness and isolation, and be associated with a lower risk of depression (Park et al., 2017). Therefore, to improve the mental health of living-alone older adults, more specific interventions should be developed and assessed, besides financial and social contact interventions.

## Limitations

This study has several limitations. First, this analysis was based on cross-sectional survey data, and causal inference cannot be drawn due to the drawbacks of the cross-sectional design. Nevertheless, the exposure (living arrangements) and the mediator (social support) were assessed based on the situation over the past 6 months to 1 year, while depression was assessed based on the situation from the last week. This temporal distinction is beneficial for estimating the mediation effect. Second, since no universal definition of living-alone was available, we used both a strict and lenient definition. Nevertheless, the results based on these definitions showed consistent conclusions. Third, the measurement of social support was not assessed by well-established scales, such as the Online Social Support Scale (Nick et al., 2018) and the Multidimensional Scale of Perceived Social Support (Canty-Mitchell and Zimet, 2000), which is insufficient and may compromise the validity of the results. Fourth, although we have adjusted for important covariables, such as age, sex, and self-reported chronic diseases, other covariables may still exist. For example, this analysis did not consider economic status and education level due to substantial missing data (74% missing for economic status and 93% missing for education level).

## Conclusions

In this national survey of a representative sample of Chinese residents aged 45 years and older, we found that low social support did not mediate the association between living status and depression. Tailored strategies for improving living arrangements may needed to improve the mental health of living alone older adults.

## Data Availability

The raw data supporting the conclusions of this article will be made available by the authors, without undue reservation.
